# Epidemiological Study of Hospital-Acquired Bacterial Conjunctivitis in a Level III Neonatal Unit

**DOI:** 10.1155/2013/163582

**Published:** 2013-05-20

**Authors:** Catarina Dias, Márcia Gonçalves, Anabela João

**Affiliations:** Unidade de Neonatologia, Centro Hospitalar de Vila Nova de Gaia/Espinho, EPE, 4400 Vila Nova de Gaia, Portugal

## Abstract

*Background*. Conjunctivitis is one of the most frequently occurring hospital-acquired infections among neonates, although it is less studied than potentially life-threatening infections, such as sepsis and pneumonia. *Objectives*. The aims of our work were to identify epidemiologic characteristics, pathogens, and susceptibility patterns of bacterial hospital-acquired conjunctivitis (HAC) in a level III neonatal unit. *Materials and Methods*. Data were collected retrospectively from patient charts and laboratory databases. Hospital-acquired conjunctivitis was defined in accordance with the Centers for Disease Control/National Healthcare Safety Network (CDC/NHSN) diagnostic criteria. *Results*. One or more episodes of HAC were diagnosed in 4,0% (*n* = 60) of 1492 neonates admitted during the study period. Most of the episodes involved premature (75,4%) and low birth weight (75,4%) neonates. Infection rates were higher among patients undergoing noninvasive mechanical ventilation (46,7%), parenteral nutrition (13,6%), and phototherapy (6,8%). Predominant pathogens included *Serratia marcescens* (27,9%), *Escherichia coli* (23%), and *Pseudomonas aeruginosa* (18%). Susceptibility patterns revealed bacterial resistances to several antibiotic classes. Gentamicin remains the adequate choice for empirical treatment of HAC in our NICU. *Conclusion*. It is important to know the local patterns of the disease in order to adjust prevention strategies. Our work contributes to the epidemiological characterization of a sometimes overlooked disease.

## 1. Introduction

Bacterial conjunctivitis is one of the most frequent hospital-acquired infections of the newborn. A few centers have recently reported the prevalence of hospital-acquired conjunctivitis (HAC) among neonatal intensive care unit (NICU) patients, despite using variable diagnostic criteria [1–4]. 

The spectrum of HAC infectious agents differs with geographic location, prophylactic ophthalmic antibiotic use, and specific health care facility microflora. In areas where universal prophylaxis is performed and where most pregnancies are adequately followed, common pathogens include *Staphylococcus aureus*, *Staphylococcus epidermidis*, *Escherichia coli,* and other Gram-negative bacteria. Coagulase-negative staphylococci are usually classified as commensal bacteria, although some reports [1–3] have listed them as the major cause of neonatal HAC.

Hospitalized newborns are particularly susceptible to bacterial conjunctivitis; their immature immune system is oftentimes challenged by adverse clinical conditions, medical devices, and invasive procedures. When left untreated, HACs can lead to severe sequelae, depending on the pathogen involved [5]. 

Surveillance is essential in order to recognize potential risk factors, help delineate prevention strategies, and adjust the antibiotics used for conjunctivitis prophylaxis and treatment. The aims of our work were to identify epidemiologic characteristics, including predisposing factors, pathogenic agents, and susceptibility patterns of bacterial hospital-acquired conjunctivitis in a level III neonatal unit.

## 2. Materials and Methods

### 2.1. Setting

 The study was performed in a neonatal unit with 6 intensive care and 12 level II care beds, with an average of 500 annual admissions. This NICU is located at a central hospital in Vila Nova de Gaia, Portugal.

### 2.2. Study Population

All neonates admitted to the NICU between January 1, 2009, and December 31, 2011, were included.

### 2.3. Data Collection

Data were collected retrospectively and consisted of demographic characteristics (e.g., gestational age, and birth weight), device utilization (mechanical ventilation, and phototherapy), procedures (e.g., surgery), parenteral nutritional support, sepsis occurrence, isolated pathogens, antibiotic susceptibility patterns, and clinical outcomes. Part of this information was only collected regarding neonates with HAC. Since all data had already been collected for clinical purposes and were available on patient charts and/or laboratory databases, the study did not require specific approval by the Ethics Committee.

### 2.4. Case-Finding Procedures

Laboratory databases were searched for all registers of ocular exudates with positive bacterial cultures. Patients were then identified and selected if HAC diagnostic criteria were met, according to the CDC/NHSN 2008 guidelines. Patients presenting two or more bacteria isolated from the same sample were also included, if these were known pathogens.

For the purpose of this study, purulent exudate samples where coagulase-negative staphylococci were the only bacteria found were not included. 

### 2.5. Microbiologic Procedures

Ocular exudates from patients conjunctivae were collected by nurses, when clinically indicated, using Amies transport swab with charcoal. Swabs were sent to the laboratory, where they were inoculated onto four bacteriological culture media: chocolate agar, MacConkey agar, Columbia colistin-nalidixic acid (CAN) agar, and chocolate agar + PolyViteX VCAT3. Plates were incubated at 35°C in 5% to 7% CO_2_, for 72 hours, and were examined daily for detection of bacterial growth. Organisms were identified by the automated system Vitek 2 and by conventional biochemical tests. Susceptibility testing was performed in accordance with the Clinical and Laboratory Standards Institute (CLSI) microdilution method.

Ocular exudates were not routinely tested for viral pathogens or *Chlamydia*.

### 2.6. Data Analysis

Data were analyzed through SPSS version 16.0, using descriptive statistics and Chi-square test, as appropriate.

## 3. Results

### 3.1. Study Population

A total of 1492 neonates were admitted during the study period. General characteristics of the population are shown in Table 1.

### 3.2. Incidence of Conjunctivitis

Hospital-acquired conjunctivitis was diagnosed in 60 (4,0%) of 1492 neonates admitted during the study period. Patients' characteristics are shown in Table 2. One of the patients presented two separate episodes of conjunctivitis during her hospital stay.

Most of the episodes concerned premature (75,4%) and low-birth weight (75,4%) neonates. Considering the study population demographics, males and females were similarly affected. There was no significant difference between groups born by cesarean section or vaginal delivery. Extreme preterms and preterms born at 28 to 31 weeks gestational age were the most affected (27,8% and 16,5%, resp.), in accordance with the conjunctivitis distribution by birth weight, which involved 35,7% of extremely low birth weight (ELBW) and 20,8% of very low birth weight (VLBW) neonates.

Almost half (46,7%) of patients who needed noninvasive mechanical ventilation (nasal continuous positive airway pressure—nCPAP) manifested an episode of conjunctivitis during their hospital stay, while HAC incidence rate among those who did not use nCPAP was much lower (2,76%). Neonates who underwent phototherapy presented an incidence rate of conjunctivitis of 6,8%, notably higher than among those without phototherapy criteria (1,8%). Conjunctivitis affected 13,6% of neonates on parenteral nutritional support and only 2,4% of those under exclusive enteral feeding. 

One patient was submitted to plastic surgery involving the face (for Pierre Robin sequence) before the onset of conjunctivitis. None of them had undergone prior ophthalmologic observation for retinopathy of prematurity or other ophthalmologic procedures.

Seven (11,7%) of the patients who developed conjunctivitis had at least one episode of sepsis during their hospital stay. Only three of these presented a common pathogen to both infections—*E. coli *in two cases and *Citrobacter koseri *in the third one.

### 3.3. Etiologic Agents and Susceptibility Patterns

The most frequently found agent was *Serratia marcescens*, comprising 27,9% of the isolates, followed by *Escherichia coli* (23%) and *Pseudomonas aeruginosa *(18%), as summarized in Table 3. Over 89% of isolated pathogens were Gram-negative bacteria. In 11,5%  (*n* = 7) of cases, more than one pathogen was found in the same exudate sample. *Serratia marcescens* was significantly more prevalent among neonates who had prior phototherapy (*P* = 0,003).

Susceptibility patterns of the most frequent bacterial isolates are summarized in Table 4.

Topical gentamicin was the antibiotic used for empirical treatment of conjunctivitis while waiting for laboratory pathogen identification and susceptibility testing. All patients had favorable outcomes.

## 4. Discussion

### 4.1. Hospital-Acquired Conjunctivitis Definition

Previous epidemiological studies have used different HAC definitions, such as one “occurring 48 hours or more after hospitalization and caused by bacterial or viral pathogens unrelated to maternal infection” [1]. According to the CDC/NHSN 2008 guidelines [6], “infections occurring in infants that result from passage through the birth canal are considered hospital-acquired infections.” Considering that conjunctivitis and other neonatal infections that become apparent within the first 28 days of life could still be acquired during birth, distinction between hospital-related and maternal origins cannot be made with certainty, especially if the isolated pathogen is common to both environments. Therefore, none of the current HAC definitions can be unequivocally applied.

Newborns admitted to the NICU few days after birth manifesting signs of conjunctivitis less than 48 hours after hospitalization were excluded from this study. Although they could still be classified as having HAC, the causative agent was probably less related to the NICU environment. 

### 4.2. Incidence  Found

Studies exclusively concerning neonates in NICUs have found cumulative incidences of endemic conjunctivitis of 5% [1], 10% [2], 11,22% [3], and 17,7% [4]. We have found a slightly lower incidence of bacterial conjunctivitis (4,1%). This might be related to the number of ocular exudates presenting coagulase-negative staphylococci (CoNS) that were not valued, to the exclusion of all exudates without an isolated pathogen or to the success of current infection control policies. Similar to other works, we did not include viral or fungal agents, nor did routinely perform investigation for *Chlamydia trachomatis* infection.

### 4.3. Isolated Pathogens

Studies conducted by Borer et al. [3], Jeong et al. [2], and Haas et al. [1] have reported CoNS as the most frequent organisms causing HAC, constituting 21 to 25% of bacterial isolates. Coagulase-negative staphylococci are not usually considered pathogenic microorganisms by our laboratory criteria, particularly if the sample is found to have only modest bacterial growth or polymorphic flora. 

Other studies have reported S. *aureus *[7], *Klebsiella *[8], and *Enterobacter *spp. [9] as the most common pathogens. We have found a predominance of Gram-negative bacteria (89%), particularly *Serratia marcescens*. This is an ubiquitous bacterium, whose complete eradication from the environment is often difficult. Nevertheless, the frequent finding of *Serratia* conjunctival isolates was not associated with an increase in *Serratia *bacteremia among our patients with HAC.

*Neisseria gonorrhoeae*, typically acquired from the colonized birth canal [10], was not found in this study. This might reflect the efficacy of neonatal ophthalmia prophylaxis performed at our hospital, although pregnant women were not routinely tested for gonococcus. Immediate postbirth ocular prophylaxis was performed with fusidic acid (1%) in 2009 and 2010 and with oxytetracycline (0.5%) in 2011.

### 4.4. Susceptibility Patterns

Most Gram-negative strains were resistant to several antibiotic classes. *Serratia*, *Pseudomonas, *and *Citrobacter *isolates had the most stable patterns, suggesting a common origin in the NICU. *Klebsiella *and *E. coli*, usually present in maternal birth canal, presented variable susceptibility patterns. We did not detect the presence of methicillin-resistant *Staphylococcus aureus*.

### 4.5. Risk Factors for HAC

According to previous studies [1, 2, 8], our work showed a higher incidence of HAC among lower birth weight and premature neonates, generally more vulnerable, as well as in the presence of known risk factors for hospital-acquired infection, such as parenteral nutrition [4]. Predominant risk factors identified in our work were phototherapy—probably related to the use of ocular protection devices—and noninvasive mechanical ventilation. Patients under nCPAP usually need more frequent manipulations. This, in association with conjunctival drying by air leaks, might contribute to an increased incidence of HAC. Impaired tear drainage through the nasolacrimal duct is another possible mechanism.

### 4.6. Limitations of This Study

Because this study used a retrospective design, we did not have sufficient data for stronger statistical analysis of different groups, concerning HAC risk factors. Other potential risk factors, such as the use of nasogastric tubes, were not investigated. 

## 5. Conclusions

Hospital-acquired conjunctivitis remains a frequent pathology among neonates. Infected conjunctivae constitute a potential reservoir for horizontal transmission of infection in the NICU if strict control measures are not adopted. Our work is an important contribution for the characterization of local microflora, although we cannot ascertain if isolated bacteria had maternal or NICU origin. It would be important to perform similar surveillance studies in other institutions, given the variability of local microbiological patterns.

Almost all the pathogens isolated were susceptible to topical gentamicin, the antibiotic of choice for empirical therapy in our NICU. This raises the question if ocular samples should be routinely sent for laboratory examination. Nonetheless, we have found four strains with intermediate gentamicin susceptibility, which alerts us for the possibility of a future increase in gentamicin-resistant bacteria.

## Figures and Tables

**Table 1:** General characteristics of the study population.

Variable	Total of patients or mean value
Gender	
Female	624 (41,8%)
Male	868 (58,2%)
Gestational age (weeks)	
28	18 (1,2%)
[28–32[	85 (5,7%)
[32–34[	93 (6,2%)
[34–37[	364 (24,4%)
≥37	932 (62,5%)
Birth weight (g)	
<750	12 (0,8%)
[750–1000[	14 (0,9%)
[1000–1500[	72 (4,8%)
[1500–2500[	464 (31,1%)
[2500–4000[	881 (59,1%)
≥4000	49 (3,3%)
Mode of delivery	
Cesarean section	721 (48,3%)
Vaginal delivery	771 (51,7%)
Mechanical ventilation	
Invasive	52 (3,5%)
Noninvasive	45 (3,0%)
Both modalities	151 (10,1%)
Phototherapy	675 (45,2%)
Parenteral nutrition	221 (14,8%)

**Table 2:** Demographics of patients presenting hospital-acquired conjunctivitis.

Variable	Total of patients or mean value
Gender	
Female	22/624 (3,5%)
Male	39/868 (4,5%)
Gestational age (weeks)	33,7 (range 26–40)
<28	5/18 (27,8%)
[28–32[	14/85 (16,5%)
[32–34[	8/93 (8,6%)
[34–37[	19/364 (5,2%)
≥37	15/932 (1,6%)
Birth weight (g)	2030 (range 795–4780)
<750	—
[750–1000[	5/14 (35,7%)
[1000–1500[	15/72 (20,8%)
[1500–2500[	26/464 (5,6%)
[2500–4000[	14/881 (1,6%)
≥4000	1/49 (2,0%)
Mode of delivery	
Cesarean section	30/721 (4,2%)
Vaginal delivery	31/771 (4,0%)
Mechanical ventilation	
Invasive	3/52 (5,8%)
Noninvasive	21/45 (46,7%)
Both modalities	7/151 (4,6%)
Previous phototherapy	46/675 (6,8%)
Parenteral nutrition	30/221 (13,6%)
Time to conjunctivitis (days)	8,5 (range 1–28)
Length of stay (days)	25,2 (range 3–88)

**Table 3:** Pathogenic bacteria causing hospital-acquired conjunctivitis.

Pathogen	*n*	%
*Serratia marcescens *	17	27,9
*Escherichia coli *	14	23,0
*Pseudomonas aeruginosa *	11	18,0
*Enterobacter cloacae *	7	11,5
*Klebsiella pneumoniae *	6	9,8
*Staphylococcus aureus *	5	8,2
*Citrobacter koseri *	3	4,9
*Haemophilus influenzae *	1	1,6
*Streptococcus pneumoniae *	1	1,6
*Enterobacter aerogenes *	1	1,6
*Proteus mirabilis *	1	1,6
*Streptococcus pyogenes* (group A)	1	1,6

**Table 4:** Susceptibility patterns of the most frequently isolated Gram-negative and Gram-positive bacteria.

Antibiotic	*Serratia marcescens *	*Escherichia coli *	*Pseudomonas aeruginosa *	*Enterobacter cloacae *	*Klebsiella pneumoniae *	*Citrobacter koseri *	*S. aureus *
Ampicillin	0/17	4/14		0/7	0/6	0/3	
Amoxicillin/clav.	0/10	4/4		0/3	1/2		
Penicillin							1/4
Oxacillin							4/4
Clindamycin							3/4
Erythromycin							4/5
Cefuroxime	0/17	5/5		0/7	0/2		
Cefotaxime	17/17	5/5		6/7	4/4	2/2	
Ceftazidime			11/11				
Cefalotin	0/17	9/12		0/7	3/6	3/3	
Gentamicin	17/17	13/14 (1)	8/11 (3)	7/7	4/6	3/3	5/5
Amikacin			3/3		2/2		
Tetracycline							3/4
TMP/SMX							4/4
Fusidic acid							3/3

Numbers inside ( ) indicate isolates with intermediate antibiotic susceptibility. TMP/SMX: trimethoprim/sulfamethoxazole.
